# Anthelmintic Activity of Extracts and Active Compounds From *Diospyros anisandra* on *Ancylostoma caninum, Haemonchus placei* and Cyathostomins

**DOI:** 10.3389/fvets.2020.565103

**Published:** 2020-09-23

**Authors:** Gabriela Janett Flota-Burgos, José Alberto Rosado-Aguilar, Roger Iván Rodríguez-Vivas, Rocío Borges-Argáez, Cintli Martínez-Ortiz-de-Montellano, Marcela Gamboa-Angulo

**Affiliations:** ^1^Departamento de Salud Animal, Facultad de Medicina Veterinaria y Zootecnia, Universidad Autónoma de Yucatán, Mérida, Mexico; ^2^Unidad de Biotecnología, Centro de Investigación Científica de Yucatán A.C., Mérida, Mexico; ^3^Departamento de Parasitología, Universidad Nacional Autónoma de México, Ciudad de México, Mexico

**Keywords:** active compounds, bioguided fractionation, gastrointestinal nematodes, plant extracts, plumbagin

## Abstract

The present study aimed to evaluate the anthelmintic activity of leaf and bark extracts of *Diospyros anisandra* collected during different seasons and their major constituents on eggs of *Ancylostoma caninum, Haemonchus placei*, and cyathostomins. Specifically, the eclosion inhibition of the methanolic extracts of the leaves and bark of *D. anisandra* collected during the dry and rainy seasons (600–37.5 μg/ml) were evaluated in addition to the fractions, sub-fractions (300–37.5 μg/ml) and active major constituents (150–2.3 μg/ml). The rainy season bark extract had the highest percentage of eclosion inhibition (PEI) against the evaluated nematodes (≥ 90% at 75 μg/ml) along with high ovicidal activity (90.0 to 93.4% at 75 μg/ml). The purification of the rainy season bark extract showed that its biological activity came from the non-polar *n*-hexane fraction (≥ 93% at 75 μg/ml). The bioguided fractionation pointed to sub-fraction 5 as having the highest anthelmintic activity against the three evaluated genera of nematodes (PEI ≥ 93% at 37.5 μg/ml). Gas chromatography and mass spectrometry revealed that the major constituent in sub-fraction 5 was plumbagin. Upon evaluation, plumbagin was confirmed to be responsible for the anthelmintic activity of *D. anisandra*, with a PEI ≥ 90% at 2.3 μg/ml on the three evaluated nematodes. Additionally, the compounds betulin and lupeol in the bark of *D. anisandra* were evaluated but presented low anthelmintic activity (PEI ≤ 5.3% at 2.3 μg/ml). In conclusion, the rainy season bark extract of *D. anisandra* exerts a high ovicidal activity against the eggs of the three studied nematodes. Plumbagin is the active compound responsible for this activity and represents a potential alternative for the control of different genera of gastrointestinal nematodes given the current scenario of anthelmintic resistance.

## Introduction

Gastrointestinal nematodes (GINs) are a serious threat to the health and well-being of domestic animals and negatively affect the economy of animal production. Also, they can negatively affect public health because of their zoonotic potential. Some examples of GINs include *Ancylostoma caninum, Haemonchus placei* and cyathostomins, which are the most prevalent and pathogenic in dogs, bovines and horses, respectively (1–4).

Traditionally, the control of GINs has been based on the intensive administration of anthelmintic drugs. However, this has generated anthelmintic resistance, mainly to benzimidazoles and macrocyclic lactones. There are also reports of *Ancylostoma caninum* resistance to pyrantel, *Haemonchus placei* resistance to salicylanilides and imidazothiazoles and cyathostomin resistance to tetrahydropyrimidines (4–7). The situation is exacerbated by multi-resistance to numerous anthelmintics, which has been documented in the aforementioned three genera (8–11).

The lack of effectiveness of current anthelmintic treatments has prompted the search for control alternatives, including the use of plant extracts with anthelmintic properties and their secondary metabolites. Plant extracts and their natural derivatives have long been used as an additional or alternative treatment to conventional chemical products and have also served as important sources of new anthelmintic molecules for the development of alternative treatments (12). Among the plants reported to have broad biological activity in the Mexican tropics is *Diospyros anisandra* (13–15). Arjona-Cambranes et al. (16) found that the bark extract of *D. anisandra* inhibited more than 98% of *Ancylostoma caninum* eclosion (the act of hatching from the egg) *in vitro* at a concentration 1,200 μg/ml and that the leaf extract showed a similar percentage of eclosion inhibition (PEI) at triple the concentration (3,600 μg/ml). Thus, the bark extract of *D. anisandra* appears to exert an ovicidal effect. Another study confirmed the effects of the methanolic extract of *D. anisandra* against cyathostomin eggs, finding a PEI > 90% at a concentration of 75 μg/ml. Specifically, two effects were noted: an ovicidal effect from using the bark extract and a larval effect using the leaf extract in which larvae failed to hatch (15).

Despite the demonstrated anthelmintic potential of *D. anisandra* in these previous studies, its extract was only evaluated in each case against one genus of nematode. The question remains as to whether *D. anisandra* extracts have a broad spectrum of action or exert effects against two or more genera of nematodes. Also, only the methanolic extracts of *D. anisandra* were evaluated without determining the active compound(s) that confers its biological activity. Hence, the objective of the present study was to evaluate the anthelmintic activity of leaf and bark extracts of *D. anisandra* collected during different seasons of the year and their major constituents on eggs of *Ancylostoma caninum, Haemonchus placei*, and cyathostomins.

## Materials and Methods

The present study was carried out at the Parasitology Laboratory of the Faculty of Veterinary Medicine (Facultad de Medicina Veterinaria) of the Autonomous University of Yucatán (Universidad Autónoma de Yucatán). The region has a warm, sub-humid climate (85.7% of the territory) characterized by a rainy (June-October) and dry (February-May) season, average annual temperature of 26°C, relative humidity between 65 and 95% and an average annual rainfall of 902 mm (17).

### Information From *Diospyros anisandra* and the Collection Site

*Diospyros anisandra* S. F. Blake is a quasi-endemic species of Peninsula of Yucatan, commonly known as k'aakalche ', k'ab che' or xanob che ', of the Ebenacea family; It can measure up to 7 meters in height, uses vary depending on the locality, such as wood, instrument making, firewood and skin diseases such as pimples, scabies and inflammation (18, 19). And it is widely distributed in the Yucatan Peninsula. *D. anisandra* is a shrub or small tree, up to 7 meters tall, short branches, glabrous. Leaves subfasciculate at the tips of the branches, obovate, rounded and reticulous at the apex, cuneate at the base, glabrous or almost glabrous, 2 to 6 cm long and 1.2 to 3 cm wide. Axillary inflorescence, from 1 to 2 staminate flowers, hanging, pedicels from 1 to 2 mm long; funelform calyx, about 4 mm long, 4 lanceolate, acuminate lobes, and about 1.5 mm long; corolla urceolada, about 14 mm long, tube 7 mm long, acuminate lobes of similar length, 1 to 2 pistillate flowers. Globose fruits, about 1 cm in diameter, black and shiny when ripe. It blooms in February (the dry season), May, June, July, and September (the rainy season), although its fruits can be seen almost all year round with the exception of April and May.

The site of collection was the municipality of Yaxcabá, wich is located in the central-southern region of the state of Yucatan, Mexico. The geographical coordinates are 20° 19' and 20° 49' north latitude and 80° 36' and 88° 56' west longitude, with an average altitude of 29 meters above sea level. The average annual temperature is 26°C, with an average annual rainfall of 1,118 mm, subject to variations due to the presence of hurricanes (20). The prevailing climate is warm sub-humid (Aw) with rains in summer, where humidity decreases from south to north with record temperatures in May and the lowest in January. The annual rainfall is 1,111 mm, with an annual relative humidity of 89%. The characteristic soil types of the municipality are Cambisols. Calcisols and in low proportion Luvisols (21). The vegetation is a sub deciduous tropical forest in different stages of succession (22).

### Plant Extracts

Leaves and bark of *D. anisandra* were collected in the rainy and dry season in Yaxcabá, Yucatán (for obtaining their extracts). Then, the aforementioned plant parts were separated and placed in a drying oven at 40°C for 48 h. The dry plant material was ground in an electric mill to reduce the size of particles to 5 mm. Two extractions were performed, each for 24 h, using methanol (MeOH) at a ratio of 30 ml for every 25 g of ground plant material. After each extraction, the methanolic solvent was separated from the plant material through filter paper and deposited in glass flasks. Using a rotary evaporator (Rotavapor, Büchi®), the solvent was eliminated under reduced pressure, concentrating the dry extract. The obtained products were placed in glass vials and stored at 4°C until use (23).

### Obtainment of GIN Eggs

Feces samples were obtained from naturally infected animals. The McMaster technique was used to determine the GINs present and quantify the number of eggs per gram of feces (24). Consecutive coprocultures were carried out to morphologically identify the L_3_ larvae of *Ancylostoma caninum* (canine), *Haemonchus contortus* (bovine) and cyathostomins (equine) (*Cyathostomum* spp type G, *Posteriostomum* spp, *Gyalocephalus capitatus Cylicoclyclus* spp type B, *Triodontophorus* spp) (25–27). Prior to processing samples for egg recovery, a centrifugal flotation of each sample was performed to ensure that eggs were in the morula stage and that larvae had not begun to form (28). The feces were macerated with purified water at a ratio of 100 ml for every 50 g of feces. The resulting mixture was filtered through non-sterile gauze and deposited in 45-ml plastic tubes, which were centrifuged at 1,500 rpm for 5 min. The supernatant was discarded, and saturated sugar solution was added (density: 1.280). The mixture was homogenized in a vortex and once again centrifuged. The eggs were recovered from the superficial portion with an inoculation loop and deposited in phosphate buffer solution (PBS). Afterwards, three washes with PBS were performed, eliminating the residues of the saturated sugar solution. The concentration of recovered eggs (eggs/ml) was estimated, and the suspension was diluted to obtain a final solution of 400 eggs/ml (15, 29).

### Egg Hatch Assay

The methanolic extracts were evaluated by hatching inhibition tests carried out according to the guidelines of the World Association for the Advancement of Veterinary Parasitology (WAAVP) (30). The evaluated concentrations were 600, 300, 150, 75, and 37.5 μg/ml. To dilute the extracts and create a negative control, a solution of PBS 0.01M (Sigma®) plus 5% absolute ethanol was used. Thiabendazole (0.1 μg/ml) was used as a positive control. Three repetitions were performed for each evaluated concentration. The extracts were diluted to the aforementioned concentrations in an ultrasonic bath (Branson®). Culture plates with 24 wells were used; in each, 0.5 ml of solution containing eggs (200 eggs approximately) was deposited in addition to 0.5 of diluted extract for a total volume of 1 ml in each well. Then, the plates were incubated in a bacteriological oven at 28°C for 48 h. At following, Lugol solution (20 μg) was added to disrupt the hatching process. The contents of each well were deposited in McMaster chambers and observed through a microscope at 10× to count the number of morulated eggs, eggs with larvae inside and hatched larvae. All plates whose negative controls obtained an eclosion percentage equal to or greater than 80% were included in the study (15, 29).

The PEI was calculated with the following formulas proposed by Peachey et al. (31) and Flota Burgos et al. (15):

$$\begin{array}{l} \;\%\;hatched=\;\left(\frac{L_{1}}{L_{1}+eggs\;}\right)\;\times \;\left(100\right)\\ \%\;egg\;hatch\;inhibition=100-\%\;hatch \end{array}$$

The effect of the extracts on larval development was calculated according to the formulas proposed by Vargas-Magaña et al. (28) and Flota Burgos et al. (15). Two effects were recorded: ovicidal activity (OA) for eggs that did not form larvae or eggs with L_1_ larvae failing eclosion (LFE) during the incubation time.

$$\begin{array}{l} \%\;OA=\left(\frac{morulated\;eggs}{morulated\;eggs+eggs\;containing\;a\;larva+L_{1}\;larvae\;}\right)\times \left(100\right)\\ \%\;LFE=\left(\frac{eggs\;containing\;a\;larva}{morulated\;eggs+eggs\;containing\;a\;larva+L_{1}\;larvae}\right)\times \left(100\right) \end{array}$$

### Fractionation of the Crude Extract of *Diospyros anisandra*

The methanolic extract of *D. anisandra* was partitioned with *n*-hexane, ethyl acetate and methanol (23). The obtained fractions (*n*-hexane, ethyl acetate and residual methanol) were evaluated by the same eclosion inhibition assays described in section Obtainment of GIN Eggs at concentrations of 300, 150, 75, and 37.5 μg/ml.

The partition with the highest anthelmintic activity was then sub-fractionated using a glass column (4 × 5 cm) with 140 g of sodic bentonite. Each sub-fraction was eluted (liquid-liquid partition) with 200 ml of *n*-hexane and then with volumes of *n*-hexane and acetone of increasing polarity (ratio of 100:0 to 0:100). Based on similar Rf values in TLC (60F254 aluminum plates coated with silica gel, Merck®) developed with the eluent *n*-hexane: acetone (8:2) and sprayed with a solution of phosphomolybdic acid, 11 fractions were grouped (23). Each sub-fraction was evaluated by hatching inhibition tests at concentrations of 300, 150, 75, and 37.5 μg/ml.

### Analysis by Gas Chromatography-Mass Spectrometry

The active sub-fraction was analyzed in a gas chromatograph (Agilent Technologies 6890N) coupled with a selective mass detector with a HP-5MS column (5% phenyl-methylpolysiloxane, 25 m × 0.2 mm internal diameter). A split injection was performed on 1 μl of a 1% solution of the F5 sample at a flow rate of 1.0 ml/min (helium as the carrier gas) and column temperature of 100°C for 3 min; then, the temperature was increased 10°C per min along a gradient until reaching a final temperature of 280°C.

The constituents in the extract were identified by searching commercial reference libraries. The fragmentation patterns of the mass spectra were compared with those in the NIST05 libraries. The major constituent (plumbagin) was then evaluated at concentrations of 150, 75, 37.5, 18.7, 9.3, and 2.6 μg/ml. Additionally, the major constituents of the bark extract of *D. anisandra*, betulin and lupeol, were isolated according to the methodology reported by Uc-Cachón et al. (32) and evaluated at concentrations of 150 to 2.6 μg/ml.

### Statistical Analysis

ANOVAs (generalized linear models) were carried out to identify significant differences between the evaluated concentrations and controls with respect to the eclosion inhibition tests (StatgraPEIcs 5.1). The lethal concentrations of the evaluated extracts were determined at 50% (LC_50_) and 99% (LC_99_) in addition to their 95% confidence intervals through Probit analysis (POLO Plus, LeOra Software®) (15).

## Results

### GIN Eclosion Inhibition by Methanolic Extracts of *D. anisandra*

The bark methanolic extract of *D. anisandra* had a greater PEI compared to the leaf extract. Considering both the bark and leaf extracts, the material collected in the rainy season (RS) had a greater PEI compared to that collected in the dry season (DS) (Table 1). The RS extract exerted the highest activity against the three genera of nematodes, with a PEI > 90% from a concentration of 75 μg/ml. For cyathostomins, the most notable effect was reached at 37.5 μg/ml (97.4%) with the RS bark extract, which was statistically similar to the PEI of thiabendazole (≥ 98.0%) (*P* > 0.05), whereas for *Ancylostoma caninum* and *Haemonchus placei*, the most notable effects were reached at 75 μg/ml, which were also statistically similar to that of thiabendazole (≥ 97.1%) (*P* > 0.05). The DS bark extracts had a PEI > 97% against *Ancylostoma caninum* and cyathostomins at 300 μg/ml and a PEI of 94.9% against *Haemonchus placei* at 600 μg/ml. Meanwhile, the leaf extract had a PEI ≥ 90% only on cyathostomin eggs, with the RS extract exerting a greater effect (98.7% at 150 μg/ml) compared to the DS extract (96.0% at 300 μg/ml).

**Table 1:** Averages and standard deviation (±) of the percentages of eclosion inhibition of the methanolic extracts of *Diospyros anisandra* against eggs of *Ancylostoma caninum, Haemonchus placei*, and cyathostomins.

**Plant part**	**Concentration (μg/ml)**	***Ancylostoma caninum***		***Haemonchus placei***		**Cyathostomins**	
		**Rainy season**	**Dry season**	**Rainy season**	**Dry season**	**Rainy season**	**Dry season**
Bark	C–	8.5 (0.9)^a^	8.6 (2.8)	1.2 (1.5)^a^	1.9 (0.5)^a^	3.4 (0.8)^a^	5.5 (1.1)^a^
	C+	98.8 (1.3)^*^^b^	99.2 (0.6)^a^	97.1 (0.8)^*^^b^	99.6 (0.5)^*^^b^	98.4 (0.9)^*^^b^	99.0 (0.1)^*^^b^
	600	99.1 (0.8)^*^^b^	97.4 (3.0)^*^^b^	99.0 (0.4)^*^^b^	94.9 (0.5)^*^^b^	99.2 (0.7)^*^^b^	98.0 (1.0)^*^^b^
	300	99.1 (0.7)^*^^b^	97.3 (0.7)^*^^b^	98.0 (1.8)^*^^b^	71.9 (7.5)^*^^c^	99.0 (0.9)^*^^b^	97.4 (1.7)^*^^b^
	150	98.8 (0.6)^*^^b^	52.8 (4.4)^*^^c^	96.7 (1.2)^*^^b^	58.9 (6.1)^*^^d^	98.8 (0.5)^*^^b^	83.9 (4.9)^*^^c^
	75	98.8 (1.2)^*^^b^	46.1 (6.2)^*^^d^	95.5 (0.1)^*^^b^	35.5 (2.9)^*^^e^	99.5 (0.7)^*^^b^	69.9 (7.7)^*^^d^
	37.5	40.4 (5.5)^*^^c^	40.9 (5.2)^*^^e^	62.2 (2.4)^*^^e^	11.3 (2.0)^*^^f^	97.4 (1.6)^*^^b^	34.5 (2.0)^*^^e^
Leaves	C–	1.0 (0.5)^a^	1.6 (1.5)^a^	1.7 (0.5)^a^	2.2 (1.9)^a^	6.4 (1.6)^a^	5.1 (1.6)^a^
	C+	99.0 (1.7)^*^^b^	99.3 (1.2)^*^^b^	99.1 (0.8)^*^^b^	99.0 (0.9)^*^^b^	99.0 (1.5)^*^^b^	97.1 (0.9)^*^^b^
	600	25.9 (0.4)^*^^c^	5.3 (0.2)^*^^c^	81.6 (2.3)^*^^c^	87.9 (8.8)^*^^c^	99.0 (0.1)^*^^b^	97.1 (1.3)^*^^b^
	300	6.7 (0.9)^*^^d^	3.0 (0.9)^d^	61.3 (1.4)^*^^d^	64.2 (8.6)^*^^d^	98.8 (1.0)^*^^b^	96.0 (1.9)^*^^b^
	150	5.8 (1.9)^*^^e^	2.9 (0.8)^e^	52.6 (0.9)^*^^e^	59.6 (5.0)^*^^e^	98.7 (0.4)^*^^b^	87.1 (2.4)^*^^d^
	75	3.6 (0.7)^*^^f^	2.7 (1.9)^f^	28.9 (0.8)^*^^f^	37.7 (1.0)^*^^f^	84.4 (1.4)^*^^c^	73.8 (4.1)^*^^e^
	37.5	1.6 (0.7)^g^	1.4 (0.4)^g^	9.2 (0.7)^*^^g^	3.5 (2.3)^g^	44.3 (5.5)^*^^d^	17.3 (2.5)^*^^f^

* *Significant differences were found with respect to the negative control*.

*Different letters among columns indicate significant differences (P < 0.05)*.

*C- Negative control; C+ Positive control*.

*Standard deviation (±)*.

*Bold values indicate Highlighted results*.

Overall, the lowest LC_50_ and LC_99_ were obtained with the RS bark extract (*P* < 0.05) (Table 2). Specifically, for cyathostomins, the lowest LC_50_ and LC_99_ were 11.3 and 38.1 μg/ml, respectively, which significantly differed from the higher lethal concentrations required by *Ancylostoma caninum* (60.0 and 76.7 μg/ml, respectively) and *Haemonchus placei* (43.1 and 128.7 μg/ml, respectively). Notably, the LC_50_ of all extracts for cyathostomins was lower than 62 μg/ml. In contrast, the LC_50_ and LC_99_ of the RS leaf extracts, DS bark extracts and DS leaf extracts were at least two times higher for all three GINs.

**Table 2:** Lethal concentrations at 50% and 99% (μg/ml) and confidence intervals (95%) of the methanolic extracts of *Diospyros anisandra* against eggs of *Ancylostoma caninum, Haemonchus placei* and cyathostomins.

**Plant part**	**Season**	***Ancylostoma caninum***		***Haemonchus placei***		**Cyathostomins**	
		**LC 50**	**LC 99**	**LC 50**	**LC 99**	**LC 50**	**LC 99**
Bark	Rainy	**60.0**^**a**^ **(52.0–66.1)**	**76.7**^**a**^ **(69.5**–**103.3)**	**43.1**^**a**^ **(27.0**–**68.0)**	**128.7**^**a**^ **(92.4**–**247.0)**	**11.3**^**a**^ **(9.1**–**13.3)**	**38.1**^**a**^ **(33.1**–**45.6)**
	Dry	132.5^ba^ (59.2–176.1)	410.3^b^ (341.0–560.1)	197.6^b^ (138.7–247.7)	694.5^b^ (583.3–890.3)	**61.2**^**b**^ **(23.5**–**84.9)**	249.6^b^ (193.6–398.4)
Leaves	Rainy	811.6^c^ (788.5–835.4)	1583.2^c^ (1524.3–1650.3)	297.5^bc^ (182.3–412.1)	1443.9^c^ (1141.5–2036.7)	**45.2**^**bc**^ **(41.2**–**48.6)**	118.4^bc^ (110.3–128.8)
	Dry	2316.3^d^ (2217.8–2.424.1)	4950.2^d^ (4676.4–5277.1)	295.4^bcd^ (147.2–444.2)	1152.6^cd^ (859.9–1912.3)	**57.3**^**bd**^ **(50.5**–**63.3)**	111.5^bcd^ (97.5–138.9)

*Different letters among columns indicate significant differences (P < 0.05)*.

*Bold values indicate Highlighted results*.

### GIN Eclosion Inhibition by Fractions and Compounds of *D. anisandra*

Based on the results obtained for the crude extracts, the RS bark extract was selected for the bioguided fractionation process. The evaluation of the fractions of the partition revealed that the active fraction with the greatest efficacy (PEI) against the three nematodes was that of *n*-hexane (PEI ≥ 90% from 75 μg/ml) (Table 3). Also, the residual methanol fraction at 600 μg/ml had a PEI of 86.6% and 59.7% against *Ancylostoma caninum* and *Haemonchus placei*, respectively. Against cyathostomins, the residual methanolic fraction and ethyl acetate showed a PEI ≥ 80% at 150 μg/ml.

**Table 3:** Average percentages of eclosion inhibition and 50% and 99% lethal concentrations (95% confidence intervals) of the products obtained from the bioguided fractionation against eggs of *Ancylostoma caninum, Haemonchus placei* and cyathostomins.

**Nematode**	**Evaluated partition**	**Percentage of eclosion inhibition (μg/ml)**	**LC_**50**_ (CI) (μg/ml)**	**LC_**99**_ (CI) (μg/ml)**
*Ancylostoma caninum*.	Methanol	80.6% (600)	418.7 (384.5–458.7)^a^	875.0 (788.4–995.5)^a^
	Ethyl acetate	3.1% (600)	ND	ND
	***n*****-hexane**	**≥** **93.7% (75)**	**62.1 (50.5**–**79.3)**^**b**^	**133.9 (107.0**–**197.0)**^**b**^
	**SF5**	**95.6 (18.7)**	**—**	**—**
*Haemonchus*	Methanol	59.7% (600)	139.6 (115.4–181.2)^c^	311.5 (246.9–454.4)^c^
*placei*	Ethyl acetate	15.5% (600)	ND	ND
	***n*****-hexane**	**≥** **98.0%** **(****31****)**	**10.9 (**–**17.0–22.5)**^**d**^	**85.3 (68.8**–**125.5)**^**bd**^
	**SF5**	**89.8 ((18.7)**	**—**	**—**
Cyathostomins	Methanol	≥ 80.0% (150)	49.5 (8.4–77.5)^de^	222.8 (170.3–353.0)^bce^
	Ethyl acetate	≥ 80.0% (150)	107.6 (92.6–119.8)^cf^	230.1 (206.5–268.5)^cdf^
	***n*****-hexane**	**≥** **96.0% (18.7)**	**3.1 (**–**0.53–5.5)**^**g**^	**22.2 (17.8**–**30.8)**^**g**^
	**SF5**	**96.7 (18.7)**	**—**	**—**

*SF5, active sub-fraction 5. CI: confidence intervals*.

*The LC_50_ and LC_99_ of sub-fraction 5 could not be determine due to the high percentages of eclosion inhibition obtained at low concentrations*.

*Bold values indicate Highlighted results*.

*Different letters among columns indicate significant differences (P < 0.05)*.

Similarly, the *n*-hexane fraction had the highest efficacy (*P* < 0.05) according to the LC_50_ and LC_99_ values (3.1 to 62.1 μg/ml and 22.2 to 133.9 μg/ml, respectively). The lethal concentrations obtained with the ethyl acetate and residual methanol fractions were at least six times higher.

The sub-fractionation of the *n*-hexane fraction showed that sub-fraction 5 had the greatest anthelmintic activity (Table 3). At 18 μg/ml, sub-fraction 5 had a PEI of 95.6, 89.8, and 96.7% against *Ancylostoma caninum, Haemonchus placei*, and cyathostomins, respectively. The remaining sub-fractions obtained a PEI of 5.9 to 11.8% against *Ancylostoma caninum*, 5.7 to 14.0% against *Haemonchus placei* and 3.3 to 18.2% against cyathostomins.

Gas chromatography-mass spectrometry revealed that the major constituent present in sub-fraction 5 was plumbagin (72.69% abundance). This compound was evaluated against the three nematode genera, obtaining a PEI ≥ 91% at 2.3 μg/ml. The LC_50_ and LC_99_ of sub-fraction 5 and its active compounds could not be determined because of the high PEI (≥ 90%) reached at all evaluated concentrations (150 to 2.3 μg/ml). In addition, the constituents betulin and lupeol found in the bark of *D. anisandra* (Table 4) were evaluated. These had low activity against the eggs of *Ancyolostoma caninum* (PEI of 3.6 and 3.2%, respectively), *Haemonchus placei* (PEI of 1.4 and 1.9%), and cyathostomins (PEI 5.0 and 5.3%) at 2.3 μg/ml. Even at the highest evaluated concentration (150 μg/ml), low PEIs were even observed against the eggs of *Ancylostoma caninum* (5.0 and 3.1%), *Haemonchus placei* (4.3 and 1.5%), and cyathostomins (8.8 and 5.5%).

**Table 4:** Average percentages of eclosion inhibition of the major constituents of the bark of *Diospyros anisandra* on *Ancylostoma caninum, Haemonchus placei* and cyathostomins (at a concentration of 2.3 μg/ml).

**Compound**	***Ancylostoma caninum***.	***Haemonchus placei***	**Cyathostomins**
Plumbagin	**91.3 (0.8)**	**92.6 (1.4)**	**92.4 (2.4)**
Betulin	3.6 (0.3)	1.4 (0.8)	5.0 (1.5)
Lupeol	3.2 (1.0)	1.9 (1.2)	5.3 (0.9)

*Bold values indicate Highlighted results*.

### Ovicidal Effect of Extracts, Fractions and Major Constituents of *D. anisandra*

**Figure 1:**
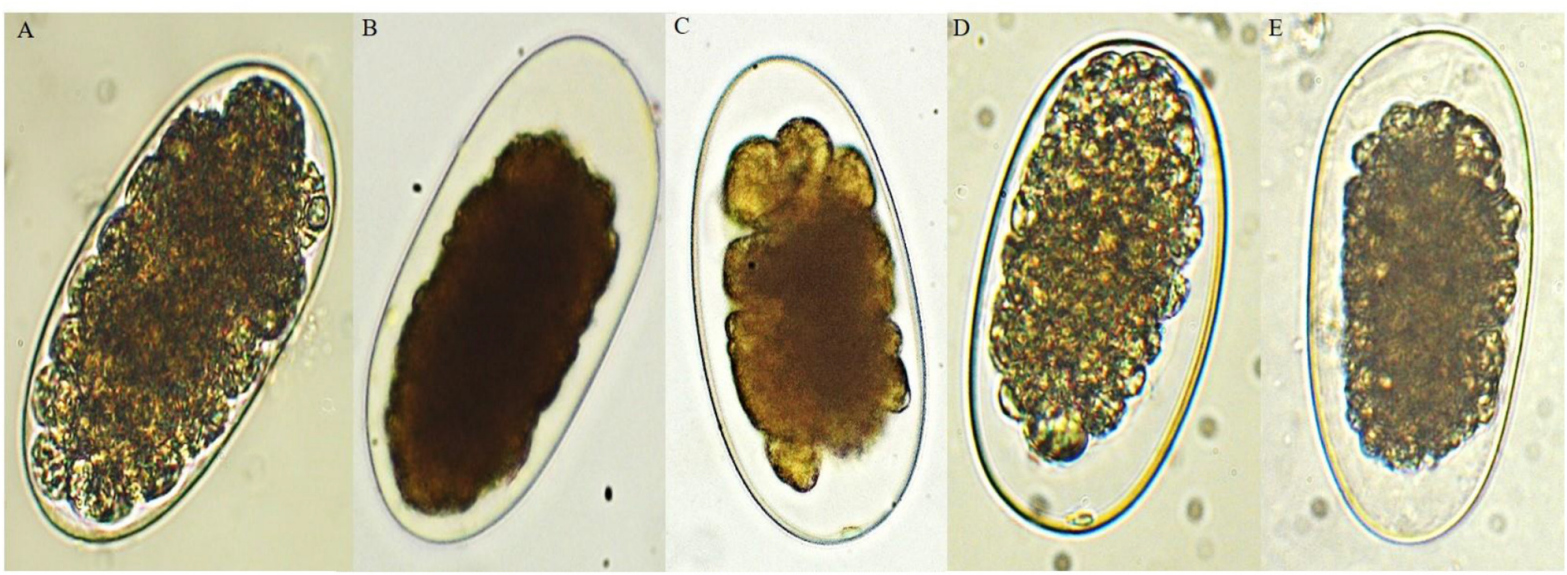
Ovicidal effect of the bark extract of *D. anisandra* collected during the rainy season (40×): **(A)** negative control with developing morula, **(B)** positive control, **(C)** *Ancylostoma caninum*, **(D)** *Haemonchus placei*, and **(E)** cyathostomins.

The methanolic extracts of *D. anisandra* bark from the RS in addition to the hexanic fraction, sub-fraction 5 and plumbagin contained within produced an ovicidal effect on the eggs of the evaluated nematodes. Inhibition of larval development was observed: After the incubation period, the morula of the eggs exposed to the extract showed signs of degeneration and a dehydrated appearance (Figure 1) (15, 28). Specifically, the RS bark extract had a percentage of ovicidal activity (POA) of 92.5, 90.0, and 95.5% at 75 μg/ml against the eggs of *Ancylostoma caninum, Haemonchus placei* and cyathostomins, respectively. At 37.5 μg/ml, it also had a high POA (92.9%) against cyathostomins. On the other hand, the DS bark extract reached a POA ≥ 86% at 600 μg/ml against *Haemonchus placei* and at 300 μg/ml against *Ancylostoma caninum*; against cyathostomins, a POA of 94.2% was reached at 300 μg/ml. Using the DS bark extract, a concentration three times was required to reach the same POA as the RS bark extract.

The POAs of the leaf extracts against *Ancylostoma caninum* were lower than 22% independently of the collection season. Against *Haemonchus placei*, a POA ≥ 50% was observed at 300 μg/ml of the leaf extracts from both seasons. However, against cyathostomins, a higher POA of 97.8% was recorded with the RS leaf extract (150 μg/ml) and 94.2% with the DS leaf extract (300 μg/ml).

**Figure 2:**
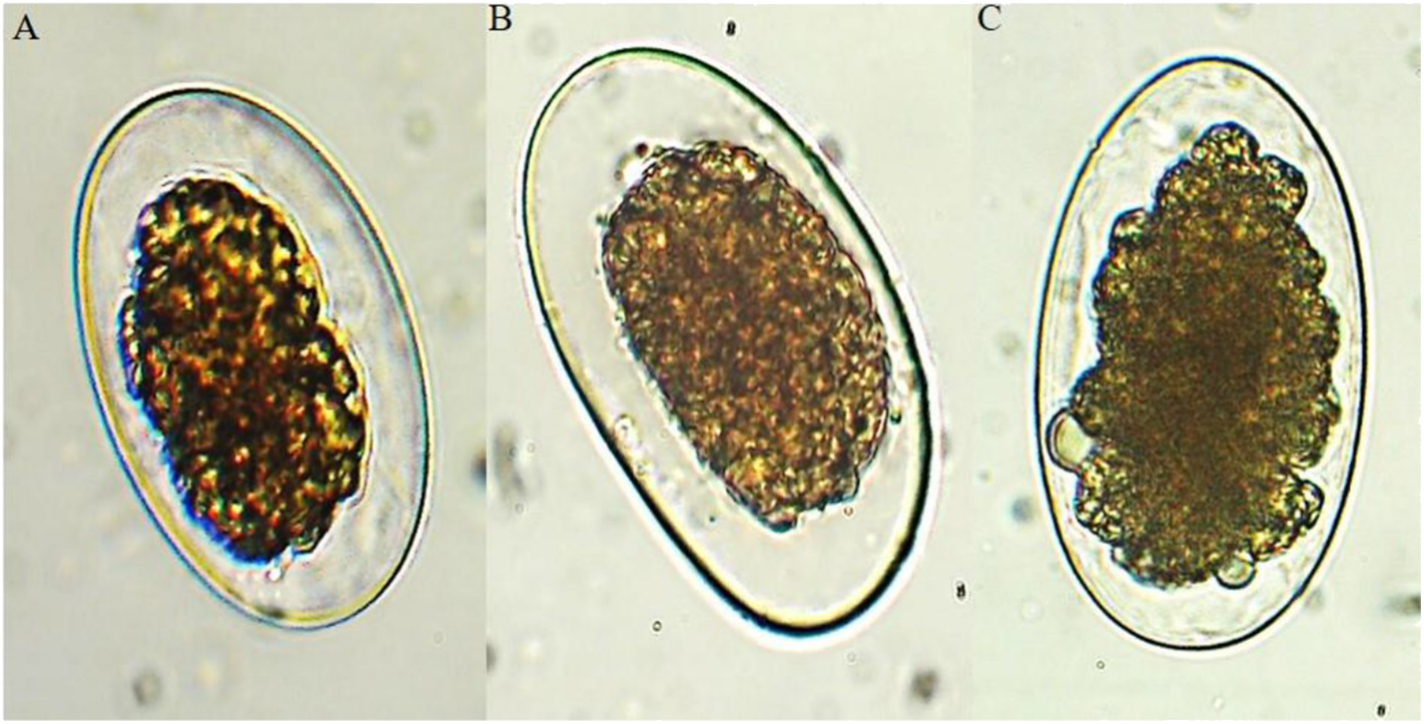
Ovicidal effect of plumbagin on eggs of the evaluated gastrointestinal nematodes (40×): **(A)** *Ancylostoma caninum*, **(B)** *Haemonchus placei*, and **(C)** cyathostomins.

The hexane fraction, fraction 5 and plumbagin only exerted an ovicidal effect on treated eggs, so the reported PEIs (Tables 3, 4) also reflect the ovicidal effect (Figure 2). With the hexane fraction, a POA of 93.7 and 98% was observed at 75 μg/ml against eggs of *Ancylostoma caninum* and *Haemonchus placei*, respectively; against cyathostomins, a similar POA (96.0%) was obtained at 18.7 μg/ml. Notably, sub-fraction 5 had a POA of 95.6, 89.8, and 96.7% against *Ancylostoma caninum, Haemonchus placei* and cyathostomins, respectively, at 18.7 μg/ml. At only 2.3 μg/ml, plumbagin had a POA of 91.3, 92.6, and 92.4% against *Ancylostoma caninum, Haemonchus placei* and cyathostomins, respectively.

## Discussion

Different plants of the genus *Diospyros* (family Ebenaceae) have been used in traditional medicine because of their high biological activity, including their antioxidant, anti-inflammatory, antipyretic, analgesic, antimicrobial, antifungal, antiprotozoal and insecticidal activities (33–36). In particular, *D. anisandra* has been attributed with numerous properties, including antimicrobial activity against multi-resistant strains of *Mycobacterium tuberculosis*; ixodicidal activity against the tick *Rhipicephalus microplus*; antiviral potential against the influenza virus; and anthelmintic activity (13–15, 32, 37).

Despite the anthelmintic potential of *D. anisandra*, previous studies have mostly focused on a single genus of GIN. So, the question remained as to whether the plant extracts of *D. anisandra* had wide-spectrum activity against two or more genera of GINs affecting different animal species. Hence, in the present study, the anthelmintic activity of *D. anisandra* was evaluated against three genera of GINs belonging to the order strongylida. Also, a modification was made to the extract dilution technique used by Arjona-Cambranes et al. (16) and Flota-Burgos et al. (15), adding 5% absolute ethanol to the solvent and using an ultrasonic bath to ensure the complete dilution of the extracts and augment their activity (38, 39).

Arjona-Cambranes et al. (16) evaluated *D. anisandra* extract against *Ancylostoma caninum* eggs and reported that the RS bark extract had a PEI of 94.1% at 1,200 μg/ml., whereas the DS bark extract had a 98.7% at 2,400 μg/ml. The leaf extracts had a PEI ≥ 90% at 2,400 μg/ml independently of the season when plant materials were collected. In contrast, in the present study, higher PEIs were obtained for a RS bark extract 15 times less concentrated (≥ 95% at 75 μg/ml) and a DS bark extract 6 times less concentrated (≥ 94% at 300 μg/ml). With respect to the RS and DS leaf extracts, a low PEI (25.9 and 5.3%, respectively) was obtained at 600 μg/ml against *Ancylostoma caninum* Notably, Arjona-Cambranes et al. (16) only used PBS as a solvent, which could have influenced the dissolution of the extracts and the obtained PEIs. Chagas (39) mentioned the importance of using suitable solvents for evaluating plant extracts *in vitro*, suggesting that an unsuitable solvent could be toxic to eggs, resulting in false positives and thereby masking the real effect of the extract. Meanwhile, (40) advised that the potential effects of plant extracts might be underestimated or discarded due to external factors, such as the poor dilution of extracts in unsuitable solvents, which could result in the rejection of potential alternative sources of anthelmintic agents.

The anthelmintic activity of other species of Diospyros or other representatives of the Ebenaceae family on eggs of *Ancylostoma* spp. has not been reported. The ethanolic extracts of *Canthium manii* (1,000 μg/ml), *Mikania laevigata, M. glomerata*, and *Euterpe edulis* (10,000 μg/ml) demonstrated a PEI of 90, 21.8, 25.9, and 21.1% (41, 42). To obtain PEIs similar to those reported in the present study, Wabo et al. (41) required at least 10 times more concentrated. On the other hand, the bark extracts of *D. anisadra* have demonstrated better results than other control alternatives for *Ancylostoma caninum* eggs, such as mushroom extracts. Hofstätter et al. (43) reported PEIs against *Ancylostoma caninum* of 59.5 to 68.3% with *Paecilomyces lilanicus* extract, 52.2 to 53.5% with *Trichoderma harzianum* extract and 56.3% with *Trichoderma virens* extract.

Several studies have evaluated the use of plant extracts for the control of *Haemonchus contortus*. For instance, the acetone extract of *Diospyros whyteana* was evaluated against *H*. *contortus* eggs and was shown to have great potential for inhibiting eclosion, with a LC_50_ range of 73.77 to 175.2 μg/ml, even though the PEI and the observed effect on eggs were not reported (44). Luka et al. (45) administered *in vivo* the ethanolic extract of *D. mespiliformis* to sheep and observed a 34.05 and 55.08% reduction in the excretion of *H. contortus* eggs at doses of 100,000 and 200,000 μg/kg, respectively. Ngaradoum et al. (46) evaluated the methanolic extract of *Ziziphus mucronate* bark, obtaining a PEI of 40 to 50% on eggs at 4,000 μg/ml. De Jesús-Martínez al. (47) examined the methanolic extracts of *Caesalpinia coraria* fruits, observing high ovicidal activity and a PEI > 98% at 780 μg/ml. Váradyová et al. (48) found that the methanolic extract of *Artemisia absinthium* had an ovicidal activity of 100% against eggs at 1,024 μg/ml. Notably, in the present study, the RS bark extract of *D. anisandra* demonstrated a high PEI (95.5% at 75 μg/ml) against *Haemonchus placei* at lower concentrations than the previous methanolic extracts.

With respect to cyathostomins, Flota-Burgos et al. (15) evaluated the methanolic extracts of *D. anisandra* collected during the RS and DS against these parasites, reporting a PEI of 95% from 37.5 μg/ml, similar to the results of the present study. No other species of *Diospyros* or member of the same family (Ebenaceae) has been reported to exert anthelmintic activity against cyathostomins. However, the anthelmintic activities of the extracts of *Acacia baileyana, A. melanoxylon, A. podalyriifolia, Alectryon oleifolius, Duboisia hopwodii, Eucalyptus gomphocephala* and *Santalum spicatum* were evaluated against cyathostomin eggs and shown to have a PEI of 100% at 1,400 μg/ml (49). Meanwhile, Peachey et al. (31) documented the anthelmintic activities of *Acacia nilotica, Cucumis prophetarum* and *Allium savitum*. Concentrations of 1,900 μg/ml were required to obtain a PEI higher than 90%. In this latter study, a high PEI (96.4%) was reported for cyathostomins at 37.5 μg/ml, around half the concentration required to reach a similar PEI for *Ancylostoma caninum* and *Haemonchus placei*. Despite the evaluated GINs belonging to the same order (strongylida) and sharing the same basic structure of the egg membrane, there is considerable variability in the thickness, composition and vulnerability of eggs, which can explain the differing degrees of susceptibility among GINs (50, 51).

It is also important to highlight that the ovicidal activity of the methanolic extracts of *D. anisandra* in the present study was similar to that of thiabendazole (52). No significant differences (*P* > 0.05) were found between the PEI of the RS bark extract at 75 μg/ml and that of thiabendazole against *Ancylostoma caninum* and *Haemonchus placei*. In the case of cyathostomins, no significant differences (*P* > 0.05) were found between the PEI of thiabendazole and that of RS and DS bark extract from 37.5 to 300 μg/ml, respectively, or the RS bark extract from 150 μg/ml and the DS leaf extract from 300 μg/ml. These results confirm that, at the aforementioned concentrations, the methanolic extracts of *D. anisandra* can reach similar efficacies as a commercial anthelmintic such as thiabendazole.

Overall, with respect to the lethal concentrations of the *D. anisandra* extracts, the lowest LC_50_ and LC_99_ were obtained with the RS bark extract. These values significantly differed (*P* < 0.05) from the lethal concentrations obtained with the DS bark extract, RS leaf extract and DS leaf extract. Arjona-Cambranes et al. (16) reported a LC_50_ and LC_99_ of 500 μg/ml and 1,700 μg/ml, respectively, for RS bark extract against *Ancylostoma caninum*, which is at least seven times higher than the values reported herein for the same extract. Specifically, a LC_50_ of 60.0, 43.1, and 11.3 μg/ml and a LC_99_ of 76.7, 128.7, and 38.1 μg/ml were found herein for *Ancylostoma caninum, Haemonchus placei*, and cyathostomins, respectively. Sakong et al. (44) reported a LC_50_ of 73.77 to 175.2 μg/ml against *Haemonchus contortus* with the acetone extract of *Diospyros whyteana*; this range is greater than that obtained herein with the RS bark extract of *D. anisandra* against *Haemonchus placei*, as well as against *Ancylostoma caninum* and cyathostomins. Meanwhile, Flota-Burgos et al. (15) documented a LC_50_ of 10.2 μg/ml with the RS bark extract of *D. anisandra* against cyathostomins, similar to the value obtained herein (LC_50_ of 11.3 μg/ml). In the present study, the LC_50_ and LC_99_ of the RS bark extract, RS leaf extract and DS leaf extract required for cyathostomins were the lowest of all the evaluated GINs; however, only the LC_50_ and LC_99_ of the RS bark extract were significantly lower. This agrees with the proposals of Bird and McClure (50) and Averlar et al. (51) concerning the structural differences and differential susceptibility of eggs, even in GINs of the same order.

In regard to the season when plant materials were collected, the RS extract had better results compared to the DS extract independently of the evaluated GIN. The concentration, composition and expression of active compounds can vary between species of the same genus, plant parts and development stages and can also be influenced by environmental factors (53, 54). Kubec and Musah (55) mentioned that the content of active compounds in plants varies depending on the climate and season of the year in which plants are collected. Valares (56) measured the flavonoid and diterpene content of the leaves and stems of *Cistus ladanifer* and found that these compounds were present in greater proportion in stems. Similarly, the total studied compounds were present in higher proportion in plants collected during the summer and in lesser proportion in plants collected during the winter. Ahmad and Mahmund (57) mentioned that plumbagin, the active compound of *Diospyros* with high biological activity, is found in greater proportion in the bark of this plant. These factors could explain the variation in PEI values and lethal concentrations obtained herein between plant parts and collection seasons.

The hexanic fraction had a higher PEI and lower LC_50_ and LC_99_ (*p* < 0.05) compared to the ethyl acetate and methanolic fractions, indicating that non-polar compounds are responsible for the activity of the bark extract of *D. anisandra* collected during the RS (Table 3). The hexanic fraction of other plants has also been reported to contain active compounds with high biological activity. For example, Rosado-Aguilar et al. (23) observed that the hexanic fraction of *Petiveria alliacea* caused the 93.6% mortality of *Rhipicephalus microplus* larvae. Other studies have shown that the compounds responsible for the biological activity of plants of the genus *Diospyros* are found in low-polarity fractions. Trongsakul et al. (33) found that the hexanic fraction of *D. variegata* Kruz exhibited a significant anti-inflammatory effect on rats in an induced oedema model as well as an antinociceptive and antipyretic effect. Meanwhile, Borges-Argáez et al. (13) reported the antifungal activity of the hexanic fraction of *D. anisandra* on *Candida albicans, Aspergillus niger*, and *Colletotrichum gloeosporioides*. Finally, Germann et al. (58) showed that the hexanic fraction of *D. kaki* lead to a notable reversion of resistance to multiple pharmaceuticals comparable to the efficacy of the positive control verapamil.

The presence of other bioactive compounds such as terpenoids, flavonoids, naphthoquinones, polyphenols, tannins, steroids, and coumarins has been reported in other plants of the genus *Diospyros* (34, 59, 60). In particular, the presence of naphthoquinones, a group of highly reactive phenolic compounds, can be highlighted. These are important for the development of new agrochemical pharmaceuticals because of their broad antiparasitic effect, which has recently generated increasing research interest (61). Also, the antimicrobial, antifungal, anti-inflammatory, anticarcinogenic, antiprotozoal and acaricidal activities of *Diospyros* have been attributed to the presence of the 1,4-naphthoquinones, specifically plumbagin (13, 32, 62–64).

Fetterer and Fleming (65) evaluated the activity of plumbagin on *H. contortus* and *Ascaris suum* and found that it inhibited 100% of the motility of L_1_ larvae at a concentration of 10.0 μg/ml; however, only 44% of egg eclosion was inhibited, with non-hatched eggs being observed as partially embryonated. Likewise, plumbagin was found to cause the death of larvae (L_1_ to L_4_) and adults of *Caenorhabditis elegans* exposed to 100 μg/ml for 24 h. Exposure to plumbagin (25 and 50 μg/ml) also had an adverse effect on the fertility of females, decreasing up to 80% the average number of eggs laid and the further development of larvae. Against eggs, plumbagin inhibited 95% of eclosion at 100 μg/ml; however, it did not present a complete ovicidal effect since eggs were still found in several stages of development, including eggs containing L_1_ larvae (66)_._ These results differ from those of the present study in which plumbagin had a PEI higher than 90% from 2.3 μg/ml against the three evaluated genera of GINs and also exerted a notable ovicidal effect on treated eggs. However, it is worth noting that the dissolution of plumbagin in the studies of Fetterer and Fleming (65) and Chaweeborisuit et al. (66) was carried out using only DMSO, possibly influencing the results and reinforcing the importance of selecting suitable solvents for *in vitro* studies. Even so, Chaweeborisuit et al. (66) observed that strains of *C. elegans* resistant to levamisole, albendazole and ivermectin presented 100% mortality after exposure to 100 μg/ml of plumbagin for 24 h, reinforcing the potential value of plumbagin as an alternative control for GINs resistant to current anthelmintics.

Other major compounds found in the bark of *D. anisandra* are the triterpenes betulin and lupeol (32). Betulin and its derivatives have been shown to have anti-inflammatory, antimicrobial, antiviral, antifungal, antimalarial, anticarcinogenic and anthelmintic activity (67–73). However, in the present study, betulin had a low PEI against the three evaluated genera of GINs (≤ 5.0%). Meanwhile, lupeol has been shown to have anti-inflammatory, antitumoral, antimicrobial and antiprotozoal activity (74–77). However, lupeol similarly had a low PEI against the three evaluated GINs (≤ 5.3%).

Similar to the methanolic extracts, the products obtained from the bioguided fractionation of *D. anisandra* herein, including plumbagin, had an ovicidal effect on the eggs of the three evaluated GINs. This finding agrees with the reports of Arjona-Cambranes et al. (16) and Flota-Burgos et al. (15), who also evaluated the ovicidal effects of *D*. *anisandra* extract on the eggs of *Ancylostoma caninum* and cyathostomins. It is important to note that several studies evaluating anthelmintic activity do not make a distinction between the type of effect observed, whether ovicidal or causing L_1_ larvae failing eclosion. However, their data are still valuable and enable us to better understand how the active compounds of *D*. *anisandra* extract exert their effects. The observed ovicidal effect suggests that the plumbagin present in the extract is capable of penetrating the membrane of a high proportion of treated eggs, damaging the morula and halting the development of larvae (40, 78). From 2.3 μg/ml, plumbagin had a similar PEI as that obtained with thiabendazole against the three evaluated GINs. Although the concentrations of the *D. anisandra* extracts that obtained a PEI similar to thiabendazole are higher than the discriminating dose, GIN resistance to benzimidazoles has been widely reported. Hence, the extracts of *D. anisandra* and plumbagin are a potential control alternative with a similar efficacy to commercially available anthelmintics. Due to their strong anthelmintic activity, future studies should examine the mechanisms of action of the methanolic extracts of *D. anisandra* and plumbagin against GIN eggs within the order strongylida and carry out *in vivo* evaluations.

In conclusion, the bark extract of *D. anisandra* collected in the rainy season had the highest anthelmintic activity against eggs of *Ancylostoma caninum, Haemonchus placei* and cyathostomins. Plumbagin was demonstrated to be the active compound responsible for the anthelmintic activity and ovicidal effect of *D. anisandra*. Because of its wide-spectrum anthelmintic activity, *D. anisandra* extract could be a potential alternative control of different genera of GINs given the current scenario of anthelmintic resistance.

## Data Availability Statement

All datasets presented in this study are included in the article/supplementary material.

## Author Contributions

JR-A, RR-V, RB-A, and GF-B proposed the study framework, designed and developed the experiment and analyzed the results. CM-O-M and MG-A participated in the experimental design and monitored the progress of the study. All authors participated in the writing of the manuscript.

## Conflict of Interest

The authors declare that the research was conducted in the absence of any commercial or financial relationships that could be construed as a potential conflict of interest.
